# Synthesis of Carbohydrate-Grafted Glycopolymers Using a Catalyst-Free, Perfluoroarylazide-Mediated Fast Staudinger Reaction

**DOI:** 10.3390/molecules24010157

**Published:** 2019-01-03

**Authors:** William Ndugire, Bin Wu, Mingdi Yan

**Affiliations:** Department of Chemistry, University of Massachusetts Lowell, 1 University Ave., Lowell, MA 01854, USA; William_Ndugire@student.uml.edu (W.N.); Bin_Wu1@student.uml.edu (B.W.)

**Keywords:** Glycopolymer, post-polymerization functionalization, perfluoroaryl azides, Staudinger reaction

## Abstract

Glycopolymers have gained increasing importance in investigating glycan-lectin interactions, as drug delivery vehicles and in modulating interactions with proteins. The synthesis of these glycopolymers is still a challenging and rigorous exercise. In this regard, the highly efficient click reaction, copper (I)-catalyzed alkyne-azide cycloaddition, has been widely applied not only for its efficiency but also for its tolerance of the appended carbohydrate groups. However, a significant drawback of this method is the use of the heavy metal catalyst which is difficult to remove completely, and ultimately toxic to biological systems. In this work, we present the synthesis of carbohydrate-grafted glycopolymers utilizing a mild and catalyst-free perfluorophenyl azide (PFPA)-mediated Staudinger reaction. Using this strategy, mannose (Man) and maltoheptaose (MH) were grafted onto the biodegradable poly(lactic acid) (PLA) by stirring a PFAA-functionalized PLA with a phosphine-derivatized Man or MH in DMSO at room temperature within an hour. The glycopolymers were characterized by ^1^H-NMR, ^19^F-NMR, ^31^P-NMR and FTIR.

## 1. Introduction

Carbohydrates are not only the core of metabolism in many biological systems, but are also integral in many cells as structural [1], communication [2], and recognition [3] elements. Synthetic carbohydrate-functionalized polymers, i.e., glycopolymers, has become an important tool in fundamental glycobiology research, and in biomedical applications such as sensing [4,5], drug delivery [6], and cryopreservation [7,8]. Synthetic glycopolymers can be categorized broadly into two types based on the mode of synthesis: (1) polymerization of carbohydrate-derivatized monomers (such as allyl [9], acrylamide [10] and acrylate [11]), and (2) grafting of carbohydrates onto a polymer backbone [12,13,14,15,16,17]. The latter technique, typically referred to as post-polymerization modification, allows facile synthesis of the polymer backbone and control over carbohydrate grafting density. To successfully graft carbohydrates onto the polymer, the conjugation reaction must be highly efficient, of high yield, and tolerant of the functional groups on the polymer. The popular ‘click’ type of reactions, particularly the copper(I)-catalyzed alkyne-azide cycloaddition (CuAAC), meet these requirements and have been applied in the synthesis of glycopolymers by post-polymerization and in glycosylation of surfaces [18,19,20]. This reaction tolerates a wide variety of carbohydrate side groups. Analytically, it offers the advantage in the form of the distinct chemical shift of the triazole proton in ^1^H-NMR that facilitates straightforward characterization [21,22]. However, a drawback of this reaction is the necessity of Cu (I) catalyst that has proved difficult to be removed completely from the products. While at least 2–5 mol% of Cu (I) is required to achieve a respectable reaction rate, even small amounts of residual metal in the final product has been shown to denature proteins and cause oxidative damage to cells [21,23,24]. Other click-type reactions that do not require metal catalysts include SPAAC (strain-promoted azide-alkyne cycloaddition), azide-aryne based “benzyne click” reaction, and iEDDA (inverse electron demand Diels–Alder) reaction [25,26,27], but these have not been applied directly in the synthesis of glycopolymers.

Another click-type reaction is the phosphine-azide Staudinger reaction [28]. The recent improvement by Bertozzi via the introduction of an ester trap [29] rendered this bioorthogonal reaction suitable for in vivo conjugations [30,31] and in oligosaccharide metabolic engineering for cell-surface labelling [29,32]. Despite its versatility, the Staudinger ligation suffers from a slow reaction rate. To overcome this issue, we have recently shown that by using an electron-deficient perfluoroaryl azide (PFPA), the rate of reaction can be increased up to four orders of magnitude to give an iminophosphorane product [33]. The rate enhancement can be attributed to the highly electronegative F atoms lowering the LUMO of PFPA thus accelerating the reactions with dienophiles and nucleophiles [34,35,36,37]. In addition, the PFPA-iminophosphorane formed in this reaction was stable and not readily hydrolyzed in vivo, as demonstrated in the metabolic labeling of A549 cells [33].

The fast reaction rate and high yield make this PFPA-Staudinger reaction an excellent candidate for polymerization. In an earlier work, we demonstrated that the reaction of bis-PFPA and a bisphosphene occurred at room temperature in 30 min to yield poly(iminophosphorane) having molecular weight of over 59,000 and a narrow dispersity of 1.1–1.2 [38]. In this work, we applied this reaction in the post-polymerization synthesis of carbohydrate–grafted glycopolymers. By taking advantage of the fast reaction rate, mild reaction conditions, and high chemoselectivity of the PFPA-Staudinger reaction, we conjugated maltoheptaose and mannose onto poly(lactic acid) (PLA), a biocompatible and biodegradable glycopolymer.

## 2. Results and Discussion

While the availability of pendant functional groups like the hydroxyl on the polymer would be suited for condensation with a carboxyl-modified sugar, this strategy is less desirable as it requires the use of coupling agents that can be difficult to remove like DCC/DMAP [39] or strong acid [40] that would hydrolyze the polymer.

**Scheme 1:**
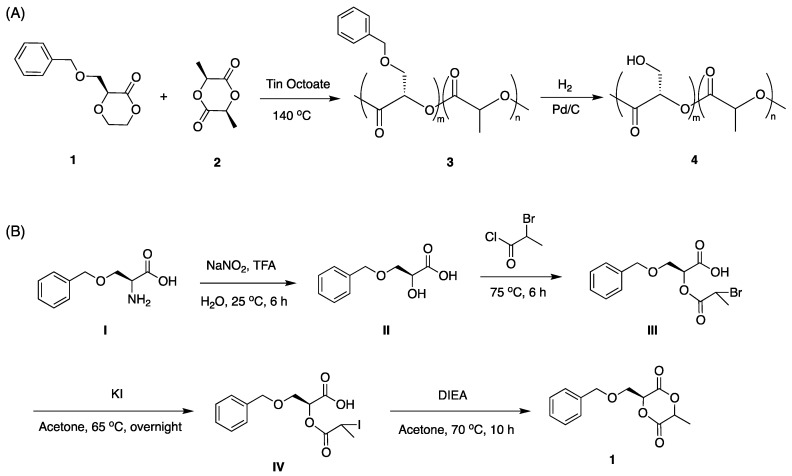
Synthesis of (**A**) hydroxy-functionalized polylactide copolymer **4**, and (**B**) lactide monomer **1**.

**Figure 1:**
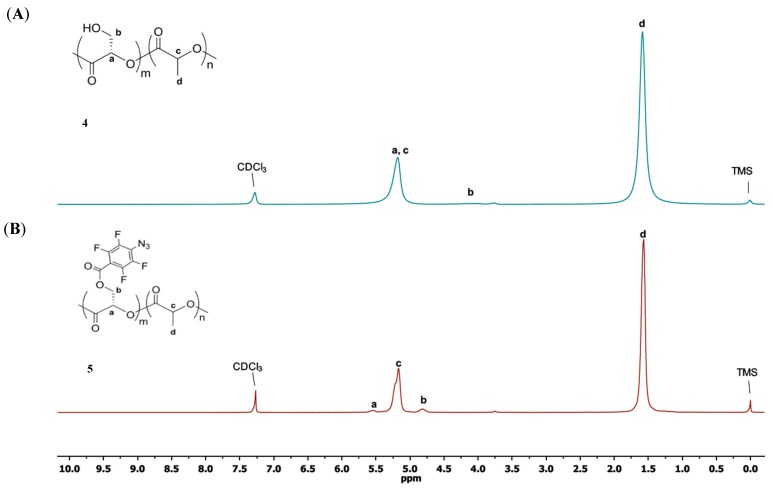
^1^H-NMR spectra of copolymers **4** (**A**) and **5** (**B**) in CDCl_3_.

Our design for grafting carbohydrates to PLA was to functionalize PLA with PFPA followed by reaction with a phosphine-derivatized carbohydrate. A PLA-*co*-PLA-PFPA copolymer is synthesized so that the density of PFPA and the carbohydrate can be varied and controlled. A hydroxy-functionalized polylactide copolymer **4** (Scheme 1A) was synthesized to conjugate PFPA and subsequently graft the carbohydrate. The benzyl-derivatized lactide monomer **1** was synthesized according to Scheme 1B. Ring-opening copolymerization of lactide **1** and lactide **2** in toluene using stannous octoate as the catalyst gave polylactide copolymer **4** in 74% yield [5]. The ratio of the two monomers can be varied so as to control the grafting density of carbohydrate on the PLA polymer. In this work, the polymer obtained had a monomer ratio (m:n) of 1:22 for **1** and **2** after copolymerization. Deprotection of the benzyl group on **3** gave the hydroxy-functionalized polylactide copolymer **4** in 63% yield [5]. The m:n ratio was calculated from the ^1^H-NMR spectrum of **4** (Figure 1A) by taking the ratio of the methine protons (H-a and H-c) that overlap at 5.2 ppm. The integral value of the H-a is equivalent to methylene protons H-b/2. Subtraction of this value from the overlapped (H-a + H-c) gives the integration of H-c and therefore H-a/H-c can be calculated.

**Scheme 2:**
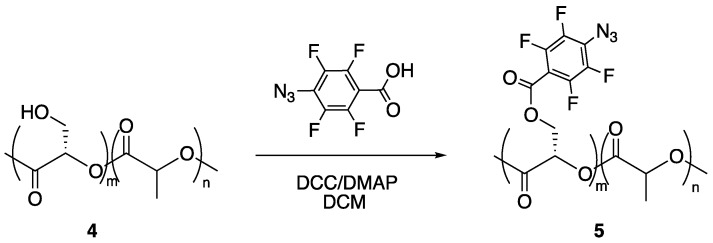
Synthesis of PFPA-PLA copolymer **5**.

PFPA-functionalized PLA copolymer **5** was prepared by esterification of **4** with carboxy-derivatized PFPA using DCC and a catalytic amount of DMAP giving **5** in 86% yield (Scheme 2).

**Figure 2:**
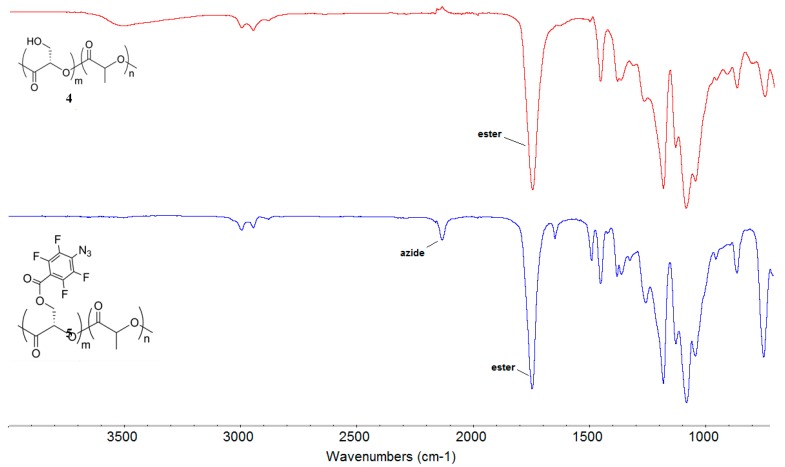
FTIR spectra of PLA copolymers **4** and **5.**

The ^1^H-NMR spectrum of **5** showed a near complete reaction as demonstrated by a marked shift of the methylene proton H-b from 4.05 ppm to 4.83 ppm (Figure 1). No degradation of the copolymers to the free lactic acid monomer was observed during the reaction, as evidenced by the absence of peaks at ~1.2 ppm which belongs to the free lactic acid. In addition, the characteristic azide peak at 2133 cm^−1^ appeared in the Fourier transform-infrared spectroscopy (FTIR) spectrum of copolymer 5 (Figure 2).

**Scheme 3:**
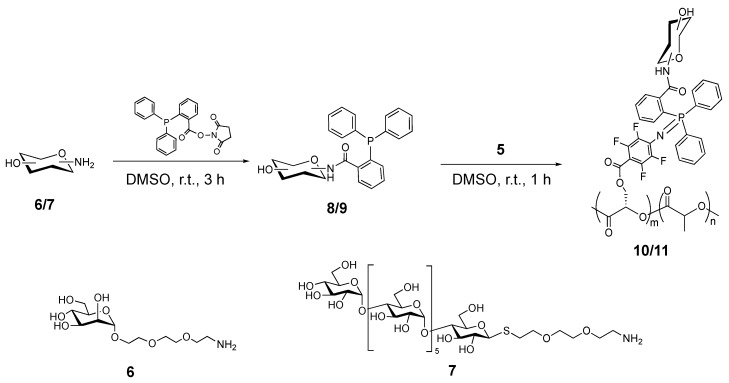
Synthesis of phosphine-derivatized mannose **8** and maltoheptaose **9**, and subsequent grafting to PFPA-PLA copolymer **5** to yield mannose-polymer **10** and maltoheptaose-polymer **11**, respectively.

The phosphine-derivatized carbohydrates were prepared from an amine-derivatized carbohydrate and the NHS-functionalized phosphine (Scheme 3). A monosaccharide, D-mannose (Man), and an oligosaccharide, d-maltoheptaose (MH), were used as model carbohydrates in this study. The amine-Man **6** was synthesized according to previously reported procedure (see Scheme S2 and detailed procedures in SI) [41]. The amine-MH **7** was synthesized following the procedure in Scheme S1 (see detailed procedures in SI). Reaction of NHS-functionalized triphenylphosphine with excess of **6** or **7** in DMSO at room temperature gave the triphenylphosphine-derivatized Man (**8**) or MH (**9**).

**Figure 3:**
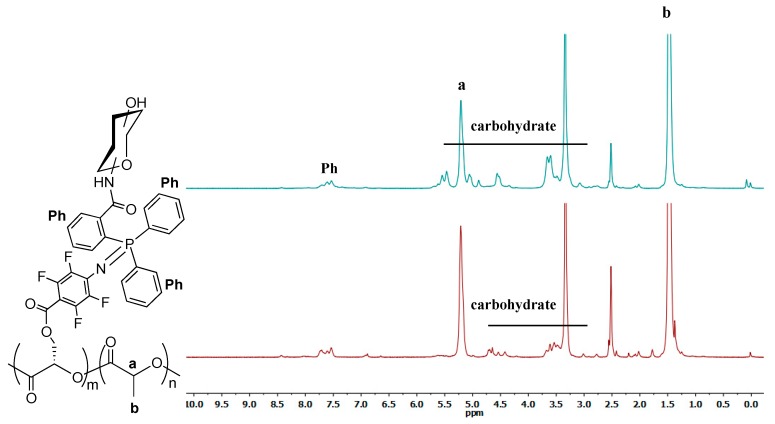
^1^H-NMR spectra of Man- and MH-grafted copolymers **10** (**top**) and **11** (**bottom**) in DMSO-d_6_.

**Figure 4:**
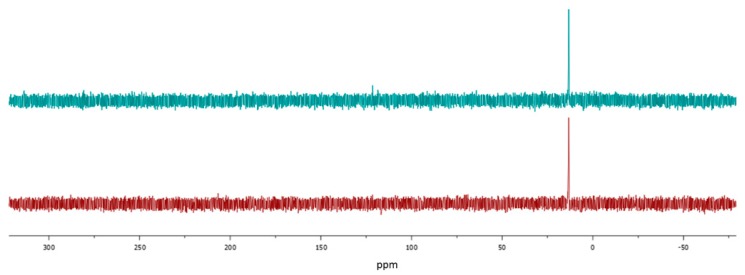
^31^P-NMR spectra of copolymer **10** (**top**) and **11** (**bottom**) in DMSO-d_6_.

To prepare Man- or MH-grafted PLA **10** and **11**, copolymer **5** was added directly to the reaction mixture of **8** or **9** in DMSO and stirred at room temperature for 1 h. The products were purified by dialysis for 48 h and dried by lyophilization. After the carbohydrate was grafted, the aromatic and the carbohydrate peaks appeared in the ^1^H-NMR spectra of **10** and **11** at 7.5–7.7 ppm and 3.0–6.0 ppm, respectively (Figure 3). In the ^31^P-NMR spectra of the products (Figure 4), a new peak was observed at 13 ppm after conjugation of the phosphine onto the copolymer. The absence of any peaks higher than 13 ppm confirmed the absence of byproducts resulting from the oxidation of phosphine.

The yield of conjugation was obtained from the ^1^H-NMR spectrum by taking the ratio of peak integration of the phenyl protons (Ph) at 7.5–7.7 ppm and the methyl protons H-b at 1.48 ppm, together with the previously calculated monomer ratio of m:n = 1:22 to give the formula:

(1) $$\left(\%\right)coupling\;yield=\left(\frac{\frac{Ph}{14}\times 22}{\frac{H-b}{3}}\right)\times 100$$

Using this equation, the yields were calculated to be 25% and 35% for the Man-grafted PLA copolymer **10** and MH-grafted PLA copolymer **11**, respectively (see Figures S5 and S6 for peak integrations).

## 3. Conclusions

In this work, we utilized an electron-deficient perfluorophenyl azide-mediated fast Staudinger reaction to efficiently synthesize carbohydrate grafted-polylactide glycopolymers by post-polymerization modification. A polylactide copolymer was synthesized by stannous octoate-catalyzed cationic ring-opening copolymerization, which was subsequently modified with PFPA. Conjugation with a phosphine-derivatized carbohydrate yield mannose- and maltoheptaose-grafted polylactide glycopolymers in 35% and 25% yields. These yields are comparable to those obtained by other groups in post-polymerization synthesis of glycopolymers using other techniques [42,43]. The main limiting factor of efficient grafting being steric hindrance between ligands and the polymer backbone restricting access to the reactive sites. In our work, this occurs between carbohydrate-phosphines reaction with the pendant PFPA groups, which is further complicated by the difference in polarity between the PLA and D-mannose/maltoheptaose. However, the grafting reaction is fast, carried out under mild conditions without the use of any catalyst. This metal catalyst-free approach to glycopolymer synthesis is significant as it eliminates the concerns over the potential toxicity of heavy metals, making these glycopolymers attractive for biomedical applications.

## 4. Experimental Procedures

### 4.1. Materials and Instruments

All chemicals and solvents were purchased from Sigma-Aldrich (St. Louis, MO, USA), TCI America (Portland, Oregon, USA) or Fisher Scientific (Hampton, NH, USA), and were used without further purification unless otherwise noted. Dichloromethane (DCM), dimethylformamide (DMF), ethyl acetate (EtOAc) and dimethyl sulfoxide (DMSO) were purified by distillation over CaH_2_. Deuterated solvents were acquired from Cambridge Isotope Lab., Inc. (Tewksbury, MA, USA). Amberlite IRC-120H^+^ resin was activated by washing with NaOH and HCl, followed by water, ethanol and toluene.

Nuclear magnetic resonance spectroscopy data were collected on either a Bruker 500 MHz spectrometer (^1^H-NMR) or a Bruker 200 MHz spectrometer (^19^F- and ^31^P-NMR) (Bruker Corporation, Billerica, MA, USA). FT-IR spectra were recorded on a Nicolet 6700 FT-IR spectrometer (Thermo Fisher Scientific, Waltham, MA, USA).

4-Azido-2,3,5,6-tetrafluorobenzoic acid (PFPA-COOH) was synthesized following our previously developed protocol [44,45]. The detailed synthesis of 1-(2-(2-(2-aminoethoxy)ethoxy)ethoxy-α-D-mannopyranoside (**6**, Scheme S2) [41,46] and 1-(2-(2-(2-aminoethoxy)ethoxy)ethoxy-maltoheptaoside (**7**, Scheme S1) can be found in the Supporting Information.

### 4.2. Synthesis of 3-benzyloxy-2-hydroxypropionic acid (***II***)

Synthesized according to literature procedure [47]. To a 200 mL of 0.7 M aqueous solution of trifluoroacetic acid, H-Ser(benzyl)-OH (**I**, 10.0 g, 52 mmol) was added, and the mixture was stirred at room temperature until all solids were dissolved. Then, 50 mL of aqueous NaNO_2_ (5.3 g, 77 mmol) was added dropwise with a syringe pump under Ar protection and the reaction was stirred for another 3 h. After confirming of the consumption of starting material by TLC, NaCl (10 g) was added and the mixture was extracted with ethyl acetate three times followed by washing with brine and dried over MgSO_4_. After passing through a flash column using CH_2_Cl_2_/MeOH/AcOH (*v*/*v*/*v* 100:8:1), compound **II** was obtained (7.2 g, 72%).^3 1^H-NMR (500 MHz, DMSO-d_6_) δ 7.35–7.26 (m, 5H, Ar-H), 4.60 (s, 2H, PhC*H_2_*O-), 4.38 (s, 1H, ‑OCH_2_C*H*(COOH)O-) 3.83 (d, *J* = 15 Hz, 2H, -OC*H_2_*CH-). IR: 3324, 3070, 3032, 2925, 2871, 2645, 2537, 1693, 1495, 1455, 1412, 1379, 1274, 1225, 1202, 1161, 1019, 1001, 973, 926, 827, 791, 732, 610, 529 cm^−1^.

### 4.3. Synthesis of 3-(benzyloxy)-2-(2-bromopropanoyloxy)propanoic acid (***III***)

Synthesized per literature [47]. Compound **II** (10.0 g, 51 mmol) and 2-bromopropionyl chloride (6.3 mL, 61.2 mmol) were mixed in a 50-mL round-bottom flask backfilled with Ar. The reaction mixture was heated to 70 °C and stirred for 6 h. Upon the completion of the reaction, the crude product was heated at 60 °C under reduced pressure to remove unreacted 2-bromopropionyl chloride and 2-bromopropionyl acid. After cooling to room temperature, the residue was washed with water and extracted with ethyl acetate 3 times. The combined organic phase was washed with brine and dried over MgSO_4_. Compound **III** was obtained as a brown oil after further purification by flash column CH_2_Cl_2_/MeOH/AcOH (*v*/*v*/*v* 100:2:0.5) (12.6 g, 75%). ^1^H-NMR (500 MHz, CDCl_3_): 7.35–7.25 (m, 5H, Ar-H), 5.34–5.32 (m, 1H, -OCH_2_C*H*(COOH)O-), 4.62–4.38 (m, 3H, PhC*H_2_*O- and -OCC*H*(Br)CH_3_), 3.97–3.85 (m, 2H, -OC*H_2_*CH-), 1.88–1.83 (m, 3H, -CH(Br)C*H_3_*). IR: 3442, 3031, 2928, 2871, 1732, 1452, 1362, 1211, 1155, 1097, 1070, 985, 910, 738, 697, 610 cm^−1^.

### 4.4. Synthesis of 3-(benzyloxy)-2-(2-iodopropanoyloxy)propanoic acid (***IV***)

Prepare following literature procedures [47]. Compound **III** (8.0 g, 24 mmol) and potassium iodide (40 g, 0.24 mol) were mixed with 100 mL of anhydrous acetone. The mixture was heated at 60 °C overnight under Ar. The solid salt was removed by passing through a layer of Celite^®^ and the filtrate was concentrated under vacuum. Ethyl acetate was added to the oily residue and the solution was filtered again to remove trace potassium iodide/potassium bromide. The organic phase was washed with 2 M aq. Na_2_S_2_O_3_ for 3 times and dried over MgSO_4_. After removing the solvent from the filtrate, the product **IV** was obtained and used directly in the next step without purification (7.5 g, 82%). ^1^H-NMR (500 MHz, CDCl_3_): δ 10.26 (s, 1H, -COO*H*), 7.50–7.16 (m, 5H, Ar-H), 5.39 (m, 1H, -OCH_2_C*H*(COOH)O-), 4.84–4.41 (m, 3H, PhC*H_2_*O- and –OCC*H*(Br)CH_3_), 3.94 (dd, J = 48.3, 10.4 Hz, 2H, -OC*H_2_*CH-), 2.29–1.87 (m, 3H, -CH(I)C*H_3_*). IR: 2923, 1728, 1496, 1452, 1362, 1197, 1124, 1094, 1043, 1026, 977, 909, 737, 697, 633, 603, 582, 570, 563, 554, 548, 538, 529, 526 cm^−1^.

### 4.5. Synthesis of 3-(benzyloxymethyl)-6-methyl-1,4-dioxane-2,5-dione (Monomer ***1***)

Synthesized as described in literature [7]. A solution of compound **IV** (10.0 g, 26.8 mmol) in dry CH_2_Cl_2_ (100 mL) was added dropwise to refluxing dry acetone (1 L) containing DIEA (8.8 mL, 53.6 mmol) under Ar. It took 10 h to finish the addition and the reaction was refluxed for another hour. The solvents were removed under reduced pressure and ether was added to dissolve the crude product. Insoluble ammonium iodide was filtered and the filtrate was concentrated. After purification by flash column chromatography using hexanes/ethyl acetate (*v*/*v* 4:1), the title compound **1** was obtained as a yellow oil (2.1 g, 31%). The diastereomers were used directly without separation. ^1^H-NMR (500 MHz, CDCl_3_): (SS) δ 7.34–7.26 (m, 5H, Ar H), 5.24–5.04 (2H; -OCC*H*(CH_2_O-)O- and - OCC*H*(CH_3_)O-), 4.61–4.56 (2H; ArC*H_2_*O-), 3.97 (2H; -OC*H_2_*CH-), 1.63 (3H; -CHC*H_3_*). IR: 2993 (w, ν_s_(aromatic C-H)), 2942 (w, ν_as_(-CH_2_- and –CH_3_)), 2872 (w, ν_s_(-CH_2_- and aliphatic -CH-)), 1748 (vs, ν_s_(ester C=O)), 1453 (m, δ_s_(-CH_2_-)), 1365 (w), 1268 (w), 1182 (s), 1084 (vs), 1046 (m), 865 (w), 739 (m, ω(aromatic C-H)), 698 (m, τ(aromatic ring)) cm^−1^.

### 4.6. Synthesis of PLA copolymer ***3***

Monomer **1** (1.0 g, 4.0 mmol), recrystallized L-lactide (**2**, 1.0 g, 6.9 mmol) and Sn(Oct)_2_ (10 mg in 1 mL anhydrous toluene) were added into a 5-mL round-bottom flask. The mixture was heated to 70 °C under vacuum for 1 h. Ar was filled in the flask and the temperature was increased to 140 °C. The mixture was stirred until the stir bar stopped moving. After cooling to room temperature, the solid was dissolved in CH_2_Cl_2_ and hexanes was added. The precipitate was re-dissolved in CH_2_Cl_2_, and this dissolution/precipitation was repeated three times, and the precipitate was finally dried under vacuum to give copolymer **1c** as a dark brown solid (1.48 g, 74%). ^1^H-NMR (500 MHz, CDCl_3_) δ 5.20 (-OCH_2_C*H*(COOH)O- and -OC*H*(CH_3_)CO-), 4.59 (PhC*H_2_*O-), 3.90 (-OC*H_2_*CH-), 1.56 (-CHC*H_3_*). IR (ATR) 2942, 1747, 1497, 1452, 1365, 1192, 1084, 1046, 865, 739, 698 cm^−1^.

### 4.7. Synthesis of PLA copolymer ***4***

Following literature synthesis [47]. Copolymer **3** (1.0 g) was dissolved in 50 mL ethyl acetate/methanol (3:1), and catalytic amount of Pd/C was added. The mixture was purged with Ar for 20 min and filled with H_2_ under vigorous stirring. After 12 h, the solution was passed through a pile of Celite to remove Pd/C and the filtrate was dried under vacuum to give copolymer **4** as a light brown solid (630 mg, 63%). ^1^H-NMR (500 MHz, CDCl_3_): δ 5.0–5.5 (-OC*H*(CH_2_OH)CO- and -OC*H*(CH_3_)CO-), 3.78 (HOC*H_2_*CH-), 1.49 (‑CHC*H_3_*). IR (ATR): ~3500 (br, w), 2995 (m), 2945 (m), 1744 (s), 1452 (m), 1380 (w), 1182 (s), 1129 (s), 1084 (s), 864 (m), 743 (m) cm^−1^.

### 4.8. Synthesis of PFPA-grafted PLA copolymer ***5***

Copolymer **4** (100 mg) and PFPA-COOH (20 mg, 0.09 mmol) were added together with DCC (41 mg, 0.2 mmol) and DMAP (2.4 mg, 0.02 mmol) to 10 mL anhydrous dichloromethane under Ar, and the mixture was stirred at room temperature overnight. The solution was then concentrated to 3 mL and was poured into 50 mL of hexane/methanol (*v*/*v* 9:1) to precipitate the crude product. The yellow precipitate was dissolved in dichloromethane and was precipitated in hexane/methanol. This dissolution/ precipitation was repeated for a total of 3 times. Finally, the precipitate was dried under vacuum to give PFPA-grafted PLA copolymer **5** as a bright yellow solid (104 mg, 86%). ^1^H-NMR (500 MHz, CDCl_3_): δ 5.56 (-OC*H*(-CH_2_OCOPFPA)CO-), 5.19 (-OC*H*(CH_3_)CO), 4.83 (‑OC*H_2_*(OCOPFPA)CH‑), 1.59 (-CHC*H_3_*). ^19^F-NMR (188 MHz, CDCl_3_): δ −137.5 (doublet), −149.62 (singlet). FTIR (ATR): 2995 (m), 2945 (m), 2133 (m), 1747 (s), 1648 (w), 1563 (m), 1490 (s), 1381 (m), 1363 (m), 1258 (s), 1182 (s), 1128 (s), 1082 (s), 1044 (s), 957 (w), 865 (m), 751 (s), 667 (m) cm^−1^.

### 4.9. Synthesis of Man- or MH-grafted PLA glycopolymers ***10*** or ***11***

#### General Procedures

The mole ratio of *N*-succinimidyl 2-(diphenylphosphanyl)benzoate:amine-carbohydrate: PFPA in polymer **10** or **11** was set as 4:6:1. *N*-Succinimidyl 2-(diphenylphosphanyl) benzoate and amine-Man **6** or amine-MH **7** were added to 5 mL anhydrous DMSO, and the solution was stirred for 3 h. Then, the mixture was added to a DMSO solution containing copolymer **5**. Afterwards, the mixture was stirred for another hour. The mixture was transferred into a dialysis tube (molecule cutoff: 3500) and dialyzed in water for 2 days. Finally, the product was obtained after lyophilization.

Polymer **10**: yellow powder (yield: 92%). ^1^H-NMR (500 MHz, DMSO-d_6_): δ 7.70–7.20 (Ar-H), 5.21 (-OCH(CH_3_)CO-), 5.80–3.00 (carbohydrate), 1.48 (-CHCH_3_); IR (ATR): 3362, 2945, 1747, 1651, 1489, 1451, 1381, 1515, 1184, 1082, 1043, 865, 749, 695 cm^−1^.

Polymer **11**: a white powder (yield: 94%). ^1^H-NMR (500 MHz, DMSO-d_6_): 7.75–7.40 (Ar-H), 5.21 (-OCH(CH_3_)CO-), 4.90–3.00 (carbohydrate), 1.48 (-CHCH_3_); IR (ATR): 3371, 2945, 1748, 1651, 1503, 1452, 1381, 1133, 1084, 865, 752, 695 cm^−1^.

## Supplementary Materials

Supporting information including detailed synthetic protocols, ^1^H, ^19^F and ^31^P NMR and IR spectra are available online.
