# Supplementary material for: Synthesis of Carbohydrate-Grafted Glycopolymers Using a Catalyst-Free, Perfluoroarylazide-Mediated Fast Staudinger Reaction
Source: Molecules. 2019 Jan 3;24(1):157. doi: 10.3390/molecules24010157 (PMC6337264; doi:10.3390/molecules24010157)
Supplement: Supplementary file 1 [file molecules-24-00157-s001.pdf]

Supporting information

Synthesis of carbohydrate-grafted glycopolymers using a catalyst-free, perfluoroaryl azide-mediated fast Staudinger reaction

*William Ndugire, Bin Wu and Mingdi Yan**

Department of Chemistry, University of Massachusetts Lowell, 1 University Ave., Lowell, MA 01854, USA

Experimental

Reagents and instruments

All chemicals and solvents were purchased from Sigma-Aldrich, TCI America or Fisher Scientific, and were used without further purification unless otherwise noted. *N*-Succinimidyl 2-(diphenylphosphanyl)benzoate[1], 4-azido-2,3,5,6-tetrafluorobenzoic acid (PFPA-COOH),[2] 2-(2-(2-azidoethoxy)ethoxy)ethanol and 1-azido-2(2-(2-iodoethoxy)ethoxy)ethane[3] were synthesized according to literature protocols.

Dry dichloromethane (DCM), dimethylformamide (DMF), ethyl acetate (EtOAc) and dimethyl sulfoxide (DMSO) were purified by distillation over calcium hydride. Amberlite® IR120 H^+ resin was activated by washing with NaOH and HCl, followed by water, ethanol and toluene.

Nuclear magnetic resonance (^{1}H NMR) data was obtained on a Bruker 500 MHz spectrometer and ^{19}F NMR data was collected from a Bruker 200 MHz spectrometer. FT-IR spectra were recorded on a Nicolet 6700 FT-IR spectrometer.

Synthesis of amine-derivatized carbohydrates

Synthesis of amine-MH S7 (i.e., 7)

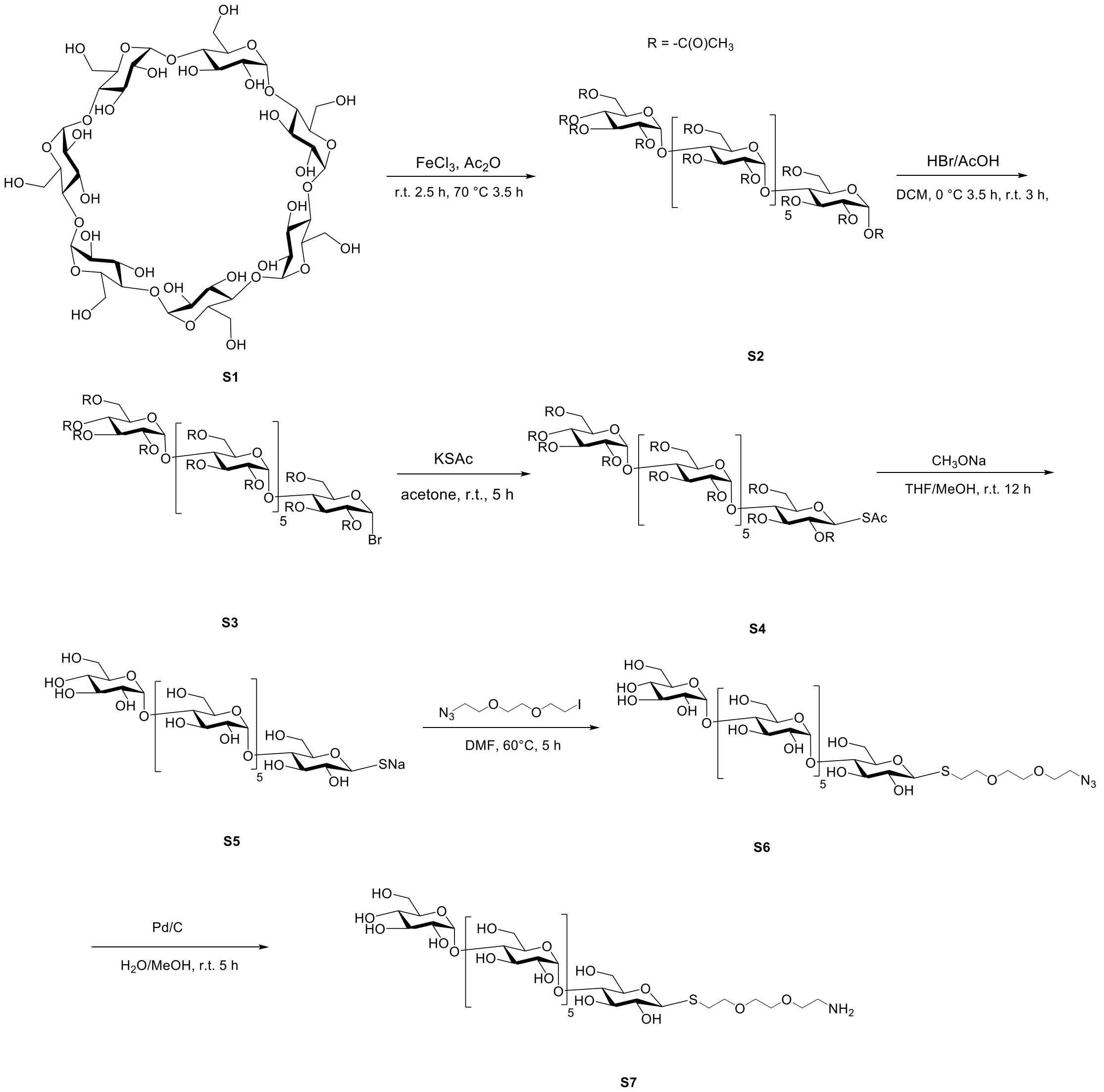

Scheme S1. Synthesis of amine-MH **S7**, R: $COCH_3$

Synthesis of tricosa-*O*-acetyl-α-D-maltoheptaose (S2)[4]

$FeCl_3$ (200 mg, 1.2 mmol) was added to Ac_2O (12.5 mL) in ice bath, and β-cyclodextrin (**S1,** 4.0 g, 3.6 mmol) was added slowly. The mixture was stirred for 2.5 h at room temperature followed by 3.5 h at 70 ^oC. After cooling to room temperature, the red solution was poured into 500 mL of water. The precipitate was collected, washed with water and cold ethanol. After recrystallization in ethanol for 3 times, tricosa-*O*-acetyl-α-maltoheptaose (**S2**) was obtained as a white solid (2.5 g, 33%). ^{1}H NMR ($CDCl_3$) δ 6.32 – 3.53 (m, 49 H), 2.44 – 1.79 (m, 69H, OAc). IR (ATR): 2961, 1739, 1433, 1368, 1212, 1163, 1026, 943, 896, 762, 636 cm^{-1}.

1-Bromo-docosa-*O*-acetyl-α-D-maltoheptaopyranose (S3)[4]

Compound **S2** (1.85 g, 0.9 mmol) was dissolved in anhydrous DCM (5 mL), cooled in an ice bath, and HBr in CH_3COOH (33% w/w, 3 mL) was added dropwise. The mixture was stirred

for 3.5 h at 0 °C, followed by 3 h at room temperature. The reaction was then diluted to 50 mL with DCM, poured into 50 mL of ice water and extracted by DCM 3 times. The combined organic phase was washed with saturated $NaHCO_3$, water and brine. After removing the solvent, compound **S3** was obtained as white solid (1.8 g, 95%). ^{1}H NMR ($CDCl_3$, 500 MHz) δ 6.49 (d, *J* = 3.6 Hz, 1H, H-1), 5.70 – 3.78 (m, 48H), 2.41 – 1.82 (m, 69H, OAc). IR (ATR): 2961, 1739, 1433, 1368, 1212, 1026, 945, 899, 763, 647 cm^{-1}.

Synthesis of docosa-*O*-acetyl-1-*S*-acetyl-1-thio-α-D-maltoheptaopyranose (S4)

Compound **S3** (1.5 g, 0.07 mmol) was dissolved in 10 mL acetone, and potassium thioacetate (239 mg, 0.21 mmol) was added under Ar. The reaction was stirred at room temperature for 5 hours. The solvent was then removed under reduced pressure and a yellow crude product was obtained. Ethyl acetate was added and subsequently washed with water 3 times followed by brine. The organic phase was dried with Na_2SO_4 and solvent was evaporated. The solid was further purified by flash column chromatography using hexane/ethyl acetate (v/v 1:3) to give compound **S4** as a light-yellow powder (1.1 g, 72%). ^{1}H NMR (500 MHz, $CDCl_3$) δ 5.47 – 5.24 (m, 14H), 5.06 (t, *J* = 9.8 Hz, 1H), 4.97 (t, 1H), 4.85 (dd, 1H), 4.78 – 4.69 (m, 5H), 4.56 – 4.44 (m, 6H), 4.37 – 4.14 (m, 7H), 4.09 – 3.84 (m, 14H), 2.38 (s, 3H), 2.26 – 1.88 (m, 66H). IR (ATR): 2961, 1739, 1434, 1368, 1212, 1163, 1027, 943, 896, 762 cm^{-1}.

Synthesis of 1-thio-β-D-maltoheptaopyranose sodium salt (S5)

To a solution of THF/methanol (v/v 1:1), compound **S4** (1.0 g, 0.48 mmol) was mixed with sodium methoxide (32 mg, 0.6 mmol) and the reaction was left stirring overnight at room temperature. The solid was centrifuge out and washed with ethanol for 5 times. Evaporation of methanol afforded **S5** as a light-yellow powder after lyophilization (0.5 g, 95%). ^{1}H NMR (500 MHz, D_2O) δ 5.42 (s, 6H), 4.57 (d, 1H), 3.99 (t, 5H), 3.93 – 3.56 (m, 35H), 3.56 – 3.50 (m, 1H), 3.45 (t, 1H), 3.07 (t,1H). IR (ATR): 3301, 2923, 1593, 1352, 1148, 1077, 1019, 931, 846, 761, 684 cm^{-1}.

Synthesis of 2-[2-(2-azidoethoxy) ethoxy]ethyl 1-thio-β-D-maltoheptaoside (S6)

Compound **S5** (1.0 g, 0.84 mmol) and 1-azido-2(2-(2-iodoethoxy) ethoxy) ethane (1.4 g, 5 mmol) were dissolved in 10 mL anhydrous DMF. The reaction was stirred for about 5 hours at 60 °C under Ar protection and monitored by TLC. After the reaction was completed, the solvent was removed under reduced pressure. The residue was passed through flash column using solvent system 1-butanol/ethanol/water (v/v/v 3:5:1) to give **S6** as a light-yellow powder (467 mg, 42%). ^{1}H NMR (500 MHz, D_2O): δ 5.30 (s, 6H), 4.52 (d, 1H), 4.00-3.20 (m, 52H), 2.90-2.60 (m, 2H). IR (ATR): 3328, 2926, 2112, 1636, 1371, 1243, 1149, 1077, 1020, 931, 848, 761, 684 cm^{-1}.

Synthesis of 2-[2-(2-aminoethoxy)ethoxy]ethyl 1-thio-β-D-maltoheptaoside (S7)

Compound **S6** (500 mg, 0.36 mmol) was dissolved in 50 mL of water and methanol mixture (v/v 8:2) under Ar protection. A catalytic amount of Pd/C (20 mg) was added under Ar protection followed by H_2 purging. The reaction was monitored by FT-IR until the azide peak at ~2100 cm^{-1} disappeared. The Pd/C was filtered out and compound **S7** was obtained as a light-yellow solid after lyophilization (455 mg, 93%). ^{1}H NMR (500 MHz, D_2O): δ 5.47 (m, 6H), 4.18 (d, 1H), 4.05-3.63 (m, 46H), 3.50 (t, 1H), 3.26 (t, 1H). IR (ATR): 3305, 2921, 1590, 1354, 1148, 1077, 1018, 931, 847, 760 cm^{-1}.

Synthesis of amine-Man S13 (i.e., 6)

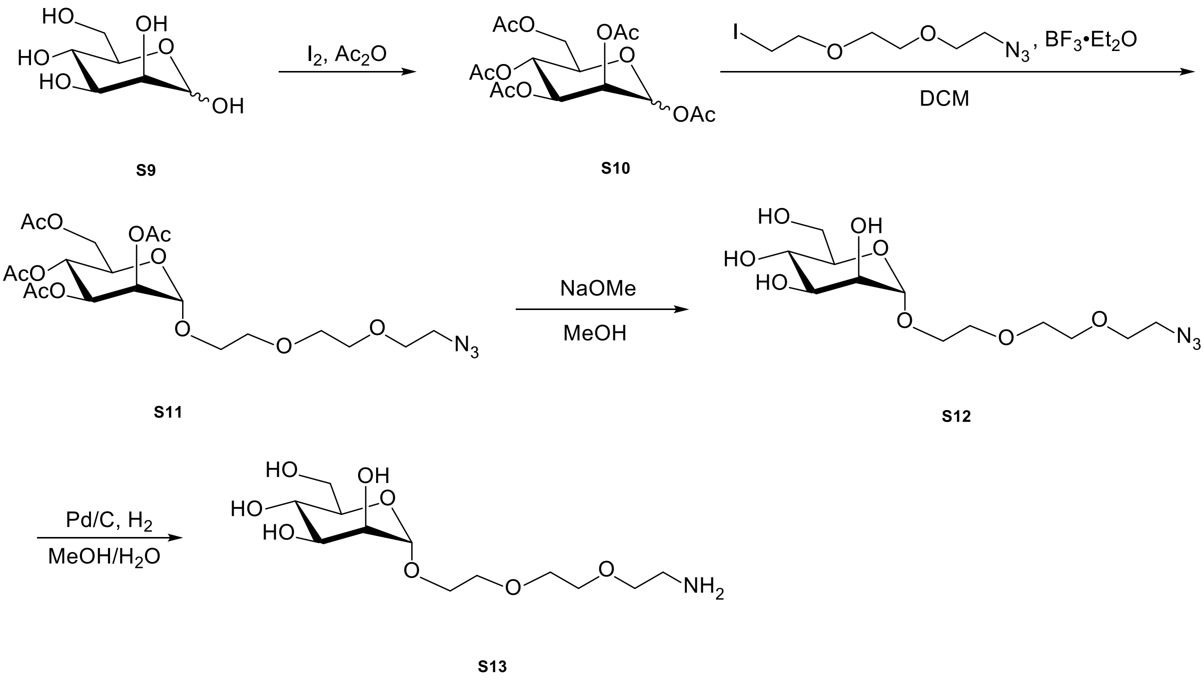

Scheme S2. Synthesis of amine-Man **S13.**

Synthesis of penta-*O*-acetyl-D-mannopyranos (S10)[5]

Iodine (50 mg, 0.2 mmol) was dissolved in Ac_2O (20 mL) and D-mannose (4.0 g, 22.3 mmol) was added slowly until all sugar was completely dissolved. After acetylation was complete, the solution was washed with cold saturated $Na_2S_2O_3$, then with 10% $NaHCO_3$ solution, after which 50 mL of DCM was added. The aqueous layer was discarded, and the organic layer was washed with water and dried over anhydrous Na_2SO_4. The solvent was removed under vacuum to give penta-*O*-acetyl-D-mannopyranose (**S10**) as a white solid (7.5 g, 88%). ^{1}H NMR (500 MHz, $CDCl_3$): (mixture of both anomers, α/β=100/73): signals of β-anomer δ 1.96-2.30 (m, 5H), 4.01–4.08 (m, 1H, 5-H), 4.10 (dd, 1H,), 4.29 (dd, 1H), 5.26 (s, 1H), 5.33–5.37 (m, 2H), 6.09 (d, 1H); signals of α-anomer δ 1.96-2.30 (m, 5H), 3.81 (ddd, 1H), 4.14 (dd,1H), 4.31 (dd, 1H), 5.14 (dd, 1H), 5.30 (t, 1H), 5.49 (s, 1H), 5.86 (s, 1H)

Synthesis of 1-(2-(2-(2-azidoethoxy)ethoxy)ethoxy-2,3,4,6-tetra-*O*-acetyl-α-D-mannopyranoside (S11)

Compound **S10** (2.0 g, 5.1 mmol) and 1-azido-2(2-(2-iodoethoxy) ethoxy) ethane (1.3 g, 7.5 mmol) were dissolved in anhydrous dichloromethane under Ar. The mixture was then cooled to 0 °C and $BF_3·Et_2O$ (3.6 g, 25.5 mmol) was injected. After overnight stirring, the reaction

mixture was washed with saturated $NaHCO_3$ and brine followed by drying over Na_2SO_4. After flash column purification (hexane/ethyl acetate (v/v 1:1)), **S11** was obtained as a viscous solid (1.2 g, 45%). ^{1}H NMR (500 MHz, $CDCl_3$): δ 5.40-5.25 (m, 3H), 4.90 (s, 1H), 4.33 (dd, 1H), 4.20-4.17 (m, 2H), 3.83 (m, 1H), 3.64 (m, 9H), 3.42 (t, 2H), 2.18 (s, 3H), 2.14 (s, 3H), 2.07 (s, 3H), 2.01 (s, 3H); IR (ATR): 2874, 2012, 1741, 1436, 1368, 1217, 1133, 1081, 1044, 978, 919, 791, 638 cm^{-1}.

Synthesis of 1-(2-(2-(2-azidoethoxy) ethoxy) ethoxy-α-D-mannopyranoside (S12)
Compound **S11** (1.0 g, 2.0 mmol) was added to 10 mL of methanol solution containing a catalytic amount of sodium methoxide (11 mg, 0.2 mmol). The mixture was stirred overnight and then neutralized by Amberlite® IR120 H^+ resin. The solvent was evaporated and **S12** was obtained as a viscous solid (634 mg, 94%). ^{1}H NMR (500 MHz, D_2O): δ 4.94 (s, 1H), 4.02 (s, 1H), 3.95-3.92 (m, 2H), 3.87 (dd, 1H), 3.81-3.65 (m, 12H), 3.57 (t, 2H); IR (ATR): 3356, 2874, 2098, 1569, 1346, 1284, 1057, 975, 880, 809, 679 cm^{-1}.

Synthesis of 1-(2-(2-(2-aminoethoxy) ethoxy) ethoxy-α-D-mannopyranoside (S13)
Compound **S12** (500 mg, 1.5 mmol) was dissolved in 50 mL of water and methanol mixture (v/v 8:2) under Ar protection. A catalytic amount of Pd/C (20 mg) was added under Ar protection followed by H_2 purging. The reaction was monitored by FT-IR until the azide peak at ~2100 cm^{-1} disappeared. The reaction mixture was then filtered through Celite®. After methanol removal and lyophilization, compound **S13** was obtained as a colorless oil (429 mg, 92%). ^{1}H NMR (500 MHz, D_2O): δ 4.84 (d, 1H), 3.92-3.90 (m, 1H), 3.85-3.80 (m, 2H), 3.79-3.74 (m, 1H, H-3), 3.72-3.51 (m, 12 H), 3.30 (s, 2H). IR (ATR): 3354, 2871, 1579, 1351, 1248, 1027, 973, 880, 806, 678 cm^{-1}.

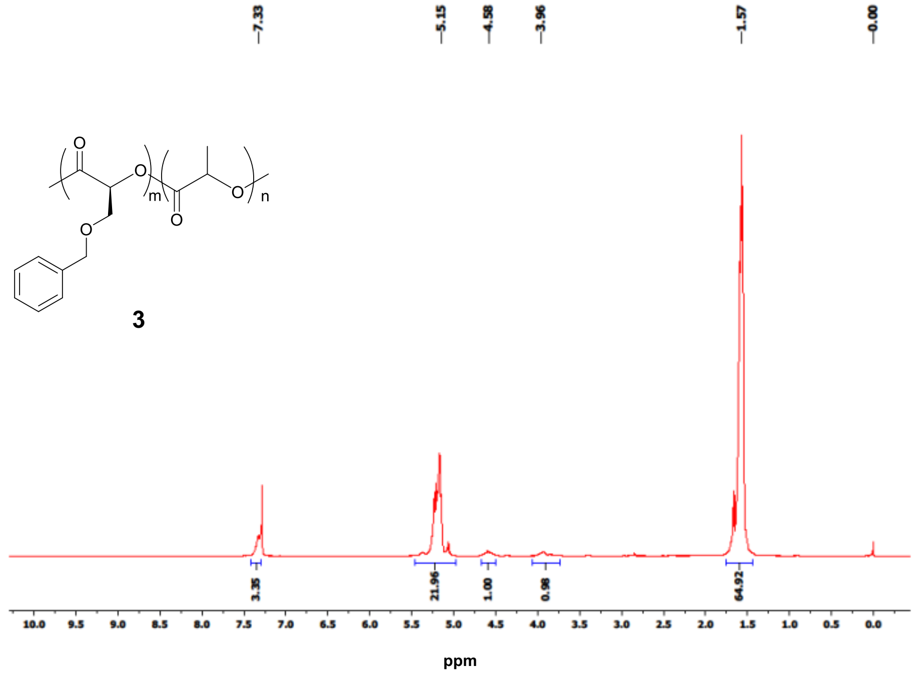

Figure S1. ^{1}H NMR of PLA copolymer **3**.

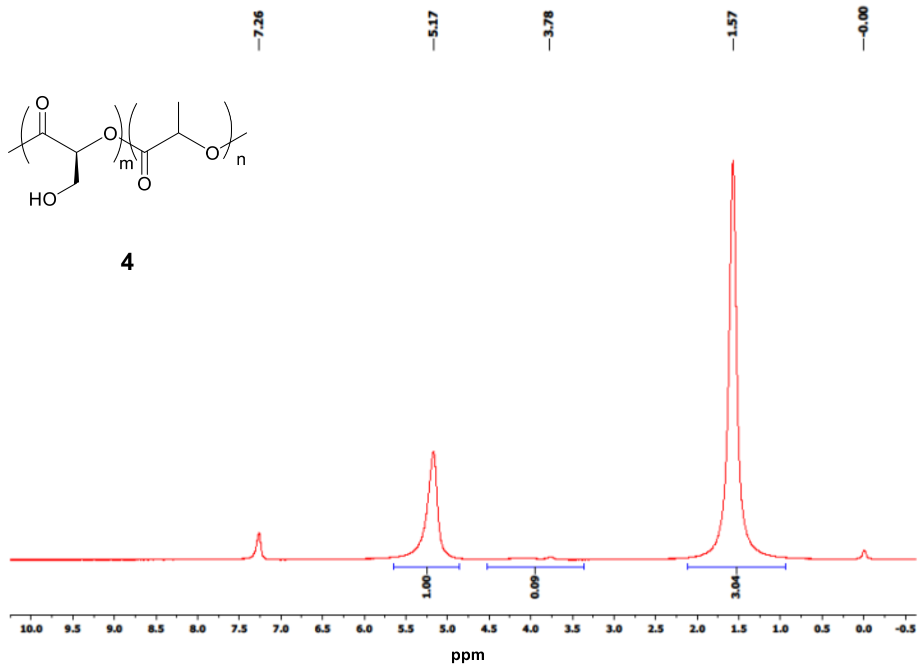

Figure S2. ^{1}H NMR of PLA copolymer **4**.

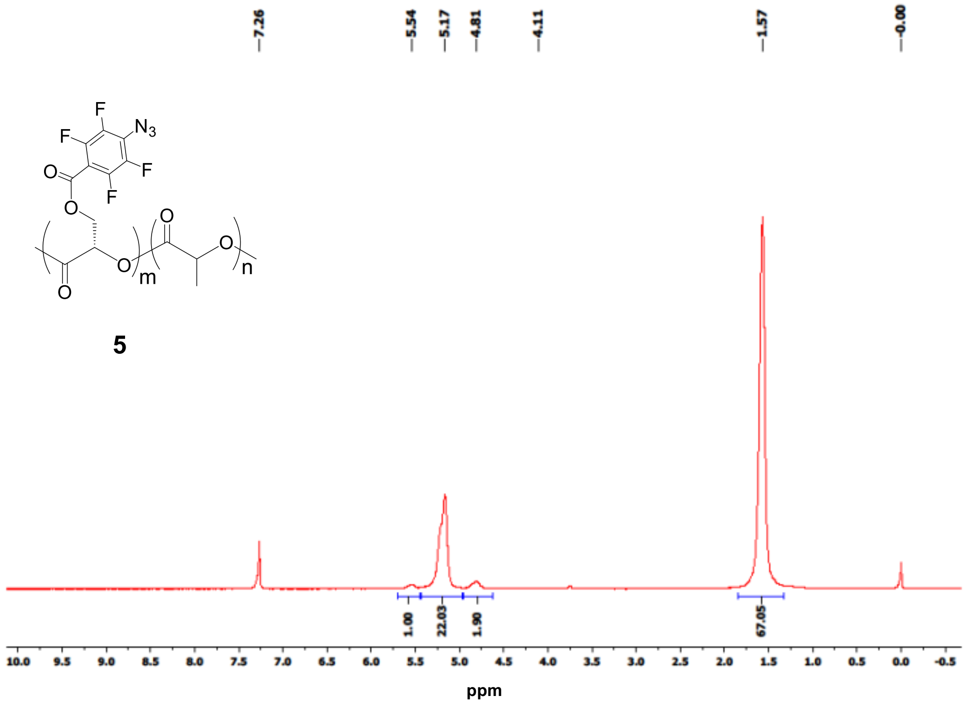

Figure S3. ^{1}H NMR of PFPA-grafted PLA copolymer **5**.

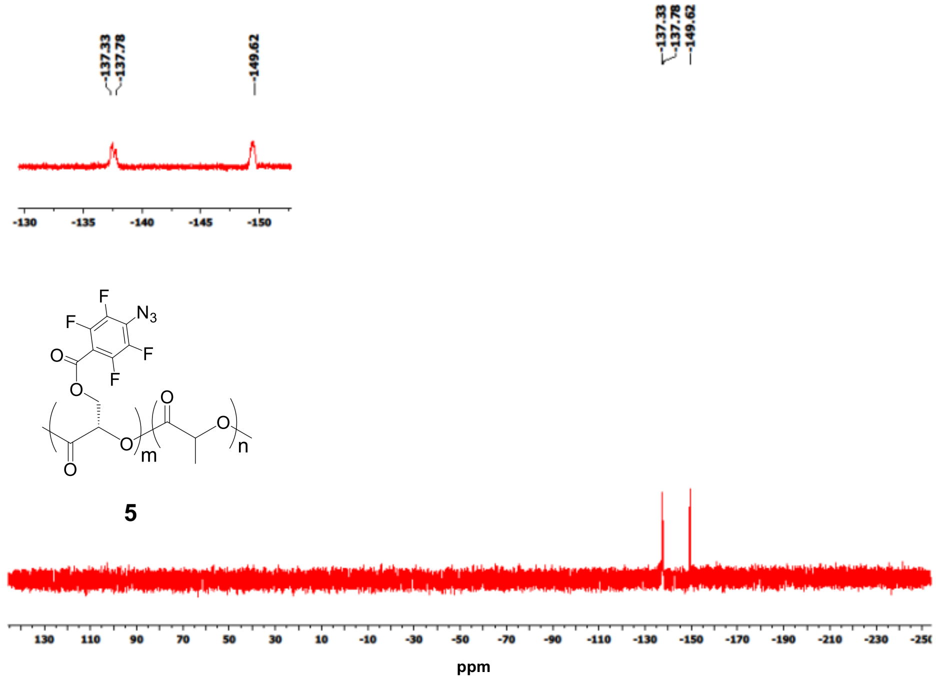

Figure S4. ^{19}F NMR of PFPA-grafted PLA copolymer **5**.

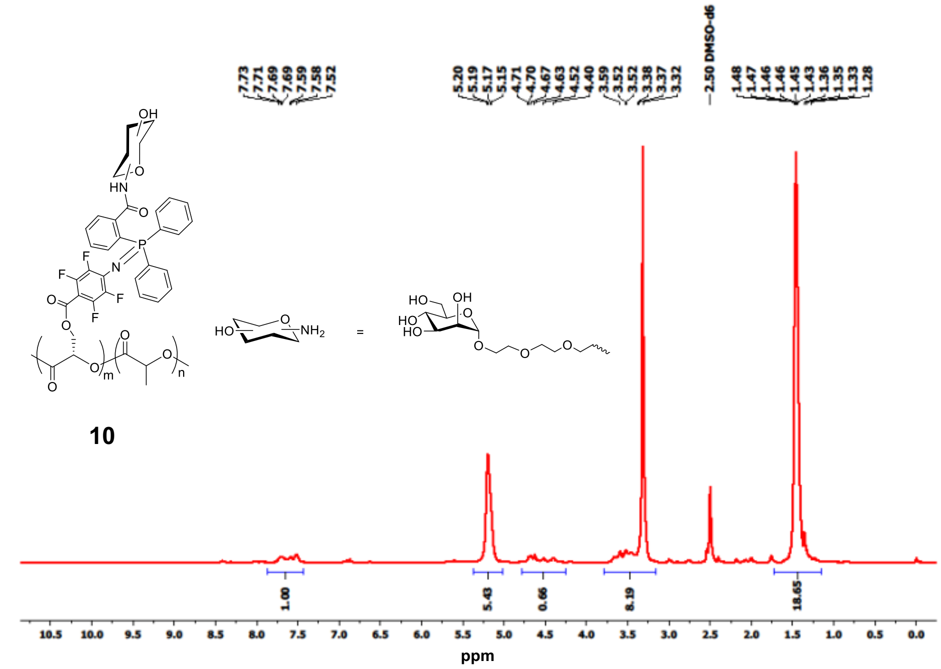

Figure S5. ^{1}H NMR of Man-grafted PLA copolymer **10**.

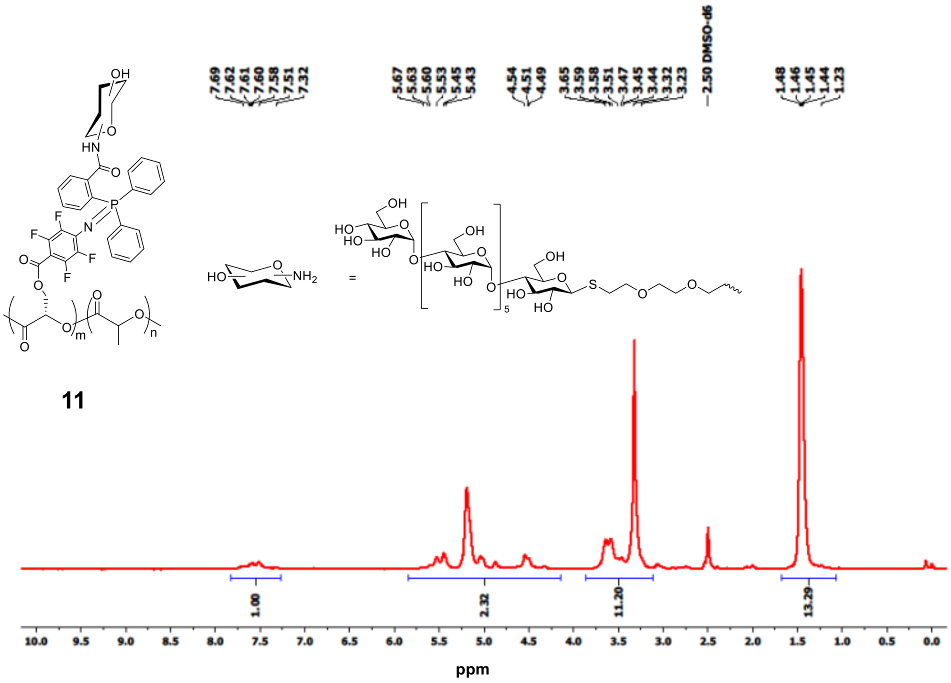

Figure S6. ^{1}H NMR of MH-grafted PLA copolymer **11**.

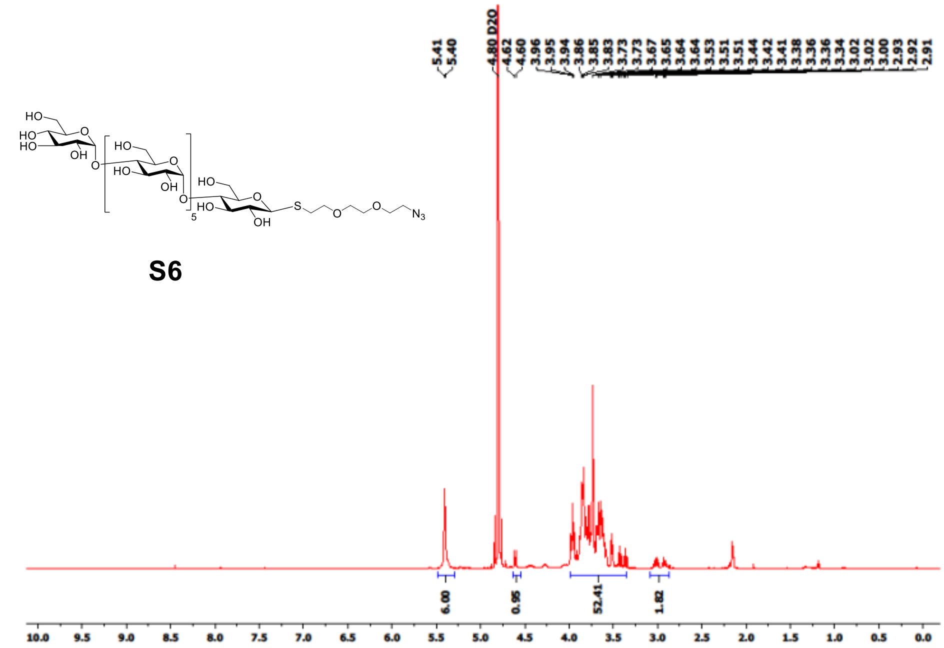

Figure S5 ^{1}H NMR of product **S6.**

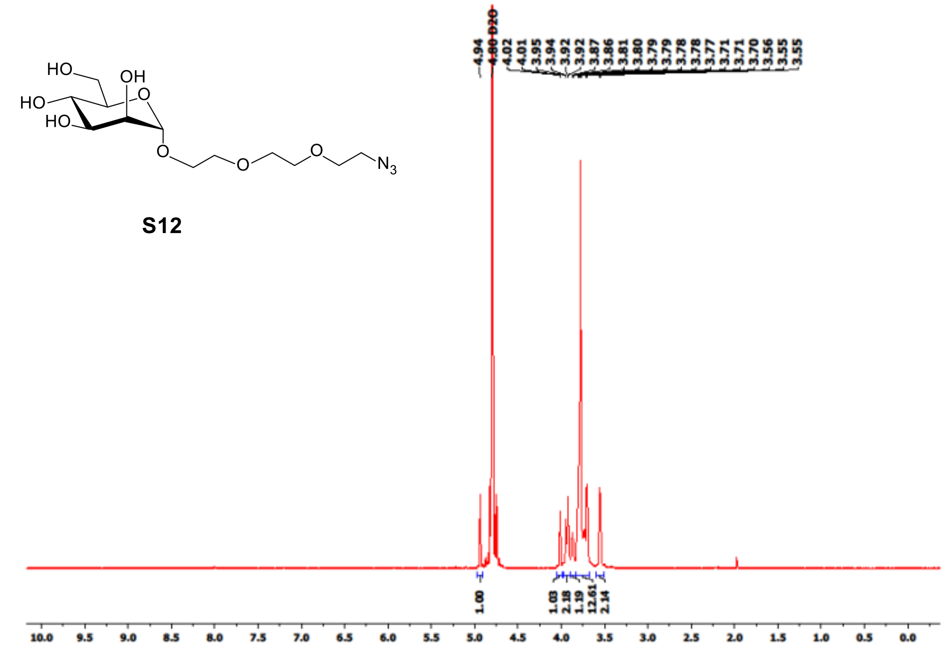

Figure S6 ^{1}H NMR of product **S12.**

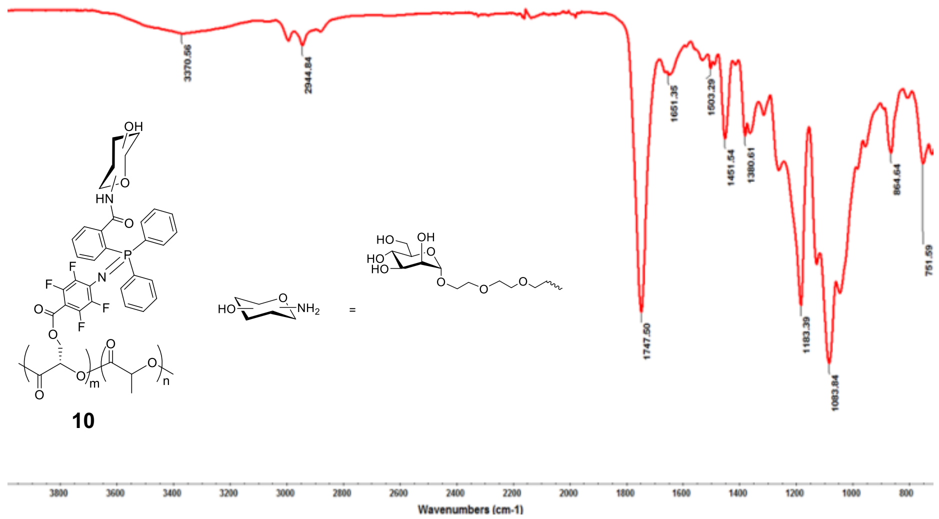

Figure S7. FT-IR spectrum of Man-grafted PLA copolymer **10**.

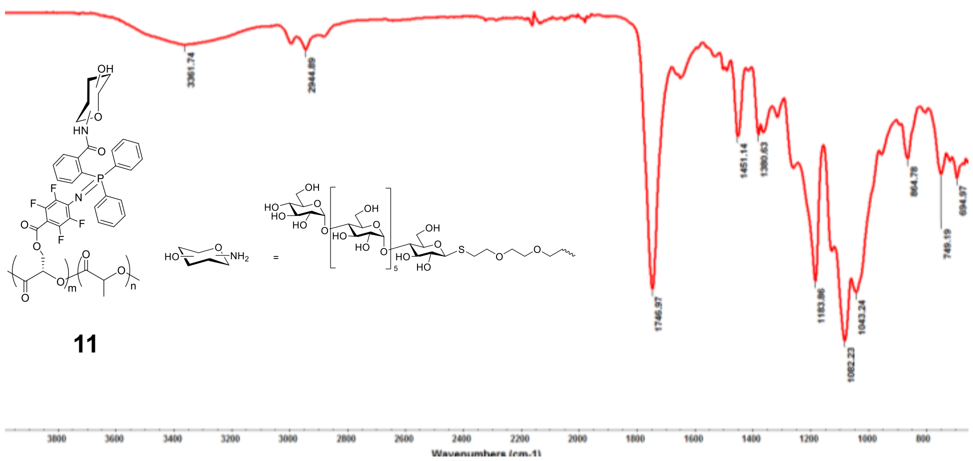

Figure S8. FT-IR spectrum of MH-grafted PLA copolymer **11**.

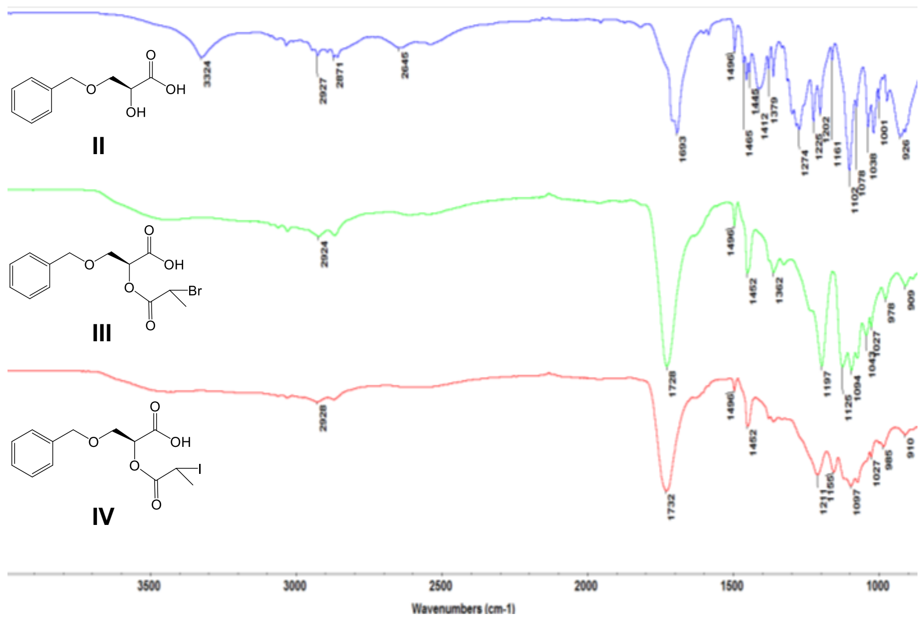

Figure S9. FT-IR spectra of products **II, III, IV.**

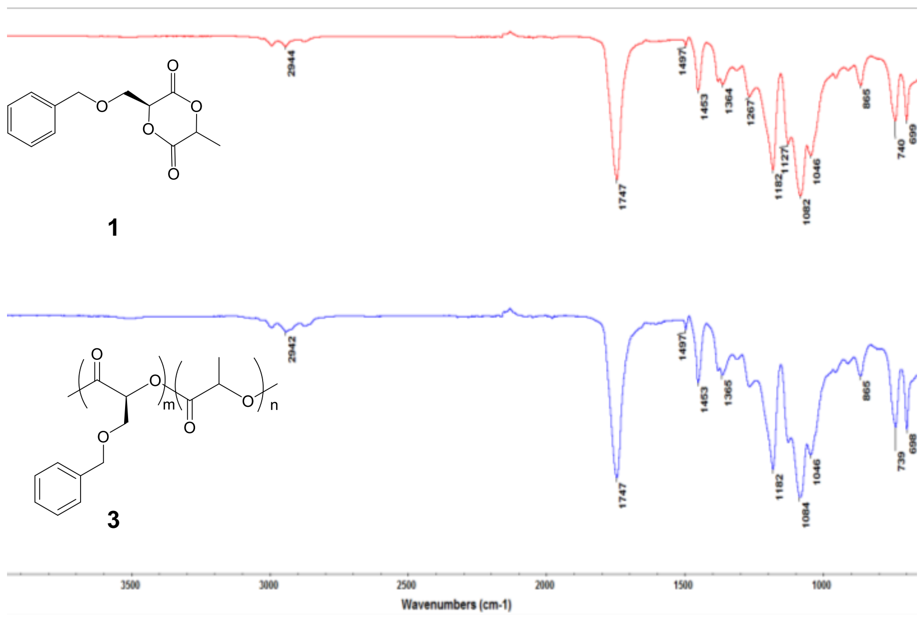

Figure S10. FT-IR spectrum of monomer **1** and the PLA copolymer **3**.

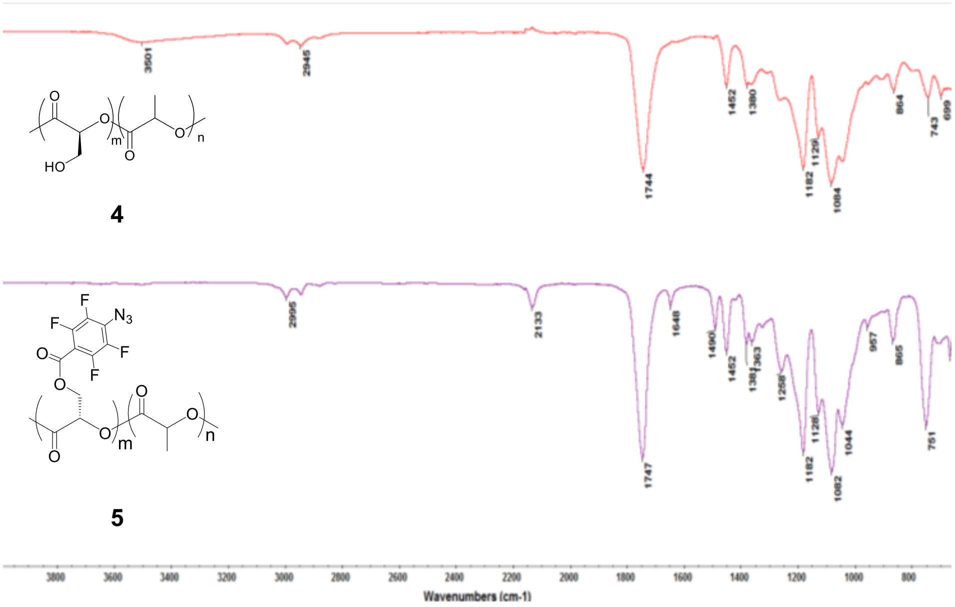

Figure S11. FT-IR spectra of PLA copolymer **4** (top) and **5** (bottom).

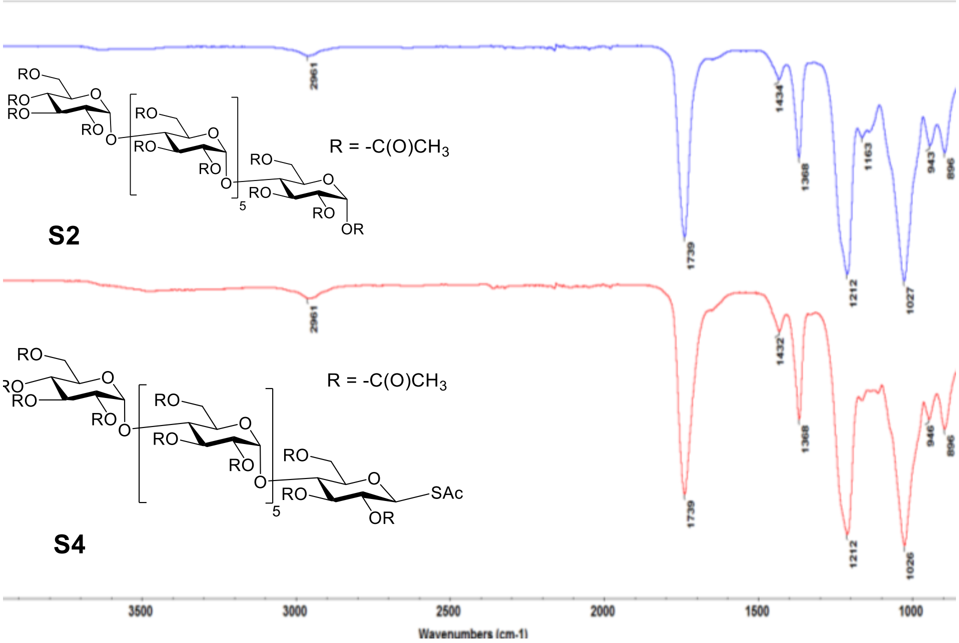

Figure S12. FT-IR spectra of compounds **S2** and **S4.**

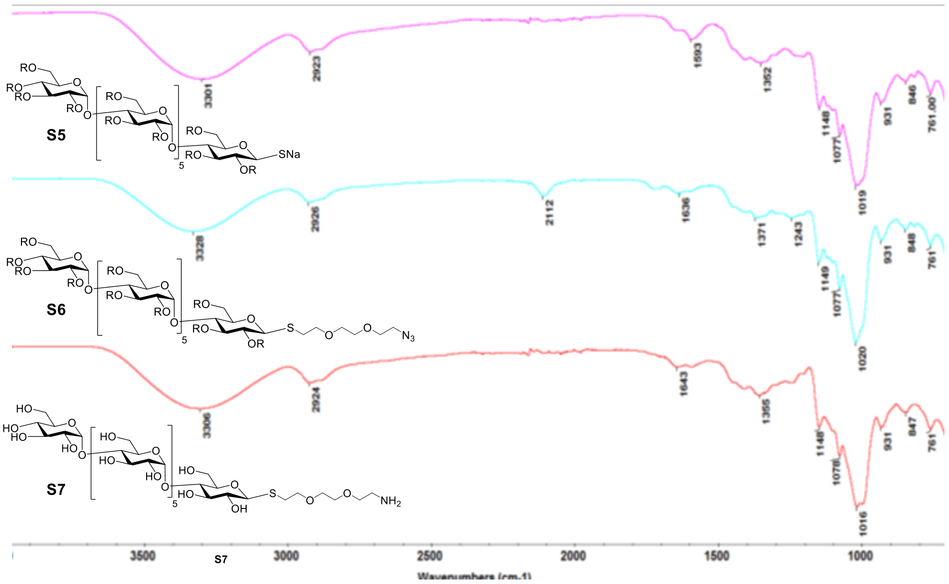

Figure S13. FT-IR spectra of compounds **S5**, **S6** and **S7.**

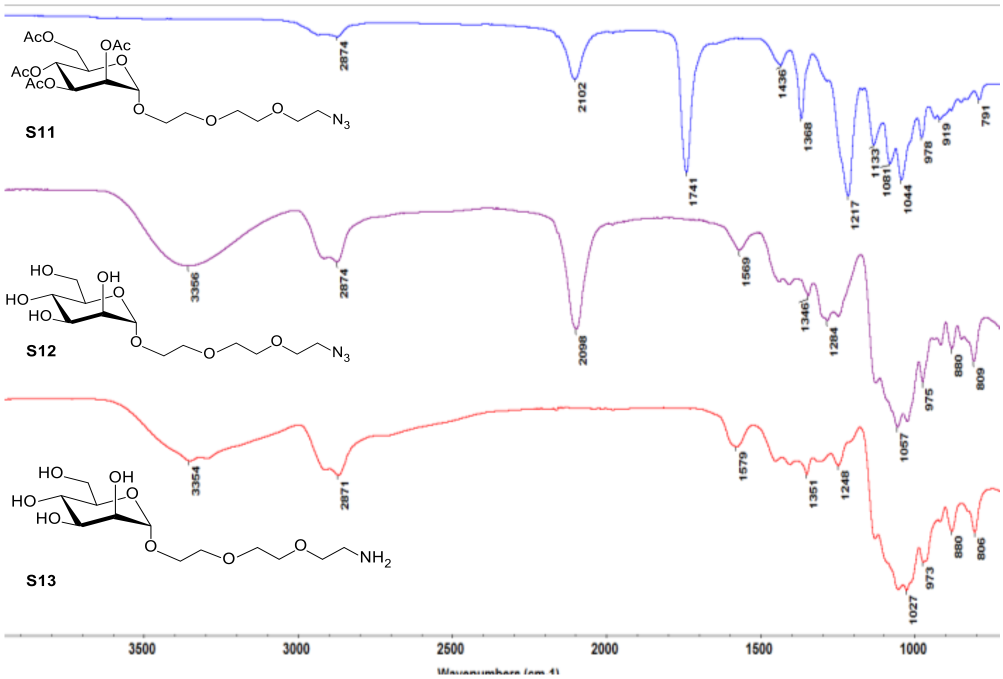

Figure S14. FT-IR spectra of compounds **S11**, **S12**, and **S13.**

References

1. Myers, E. L.; Raines, R. T., *Angew. Chem., Int. Ed.* **2008,** *48* (13), 2359-2363.
2. Yan, M.; Ren, J., *Chem. Mater.* **2004,** *16* (9), 1627-1632.
3. Deng, L.; Norberg, O.; Uppalapati, S.; Yan, M.; Ramstrom, O., *Org. Biomol. Chem.* **2011,** *9* (9), 3188-3198.
4. Farkas, E.; Jánossy, L.; Harangi, J.; Kandra, L.; Lipták, A., *Carbohydr. Res.* **1997,** *303* (4), 407-415.
5. Hayes, W.; Osborn, H. M. I.; Osborne, S. D.; Rastall, R. A.; Romagnoli, B., *Tetrahedron* **2003,** *59* (40), 7983-7996.
